# On the effects of memory and topology on the controllability of complex dynamical networks

**DOI:** 10.1038/s41598-020-74269-5

**Published:** 2020-10-15

**Authors:** Panagiotis Kyriakis, Sérgio Pequito, Paul Bogdan

**Affiliations:** 1 Ming Hsieh Department of Electrical and Computer Engineering, University of Southern California, Los Angeles, CA 90007 USA; 2 Department of Industrial and Systems Engineering, Rensselaer Polytechnic Institute, Troy, NY 12180 USA

**Keywords:** Engineering, Computer science

## Abstract

Recent advances in network science, control theory, and fractional calculus provide us with mathematical tools necessary for modeling and controlling complex dynamical networks (CDNs) that exhibit long-term memory. Selecting the minimum number of driven nodes such that the network is steered to a prescribed state is a key problem to guarantee that complex networks have a desirable behavior. Therefore, in this paper, we study the effects of long-term memory and of the topological properties on the minimum number of driven nodes and the required control energy. To this end, we introduce Gramian-based methods for optimal driven node selection for complex dynamical networks with long-term memory and by leveraging the structure of the cost function, we design a greedy algorithm to obtain near-optimal approximations in a computationally efficiently manner. We investigate how the memory and topological properties influence the control effort by considering Erdős–Rényi, Barabási–Albert and Watts–Strogatz networks whose temporal dynamics follow a fractional order state equation. We provide evidence that scale-free and small-world networks are easier to control in terms of both the number of required actuators and the average control energy. Additionally, we show how our method could be applied to control complex networks originating from the human brain and we discover that certain brain cortex regions have a stronger impact on the controllability of network than others.

## Introduction

In recent years, there has been an interplay between network science and control theory mainly due to the fact that many natural and man-made systems, such as the power grid, the World-Wide-Web, neural and biological networks can be described by complex dynamical networks (CDNs). A CDN consists of two components: the network component and the dynamical component. On the one hand, the network component has been extensively studied in the past two decades, strongly stimulated by the exploration and advance of small-world networks (Watts–Strogatz model^1^) and scale-free networks (Barabási–Albert model^2^), both of which are considered developments that follow the notion of the classical Erdős–Rényi random graph model^3^. On the other hand, the indispensable dynamical component is required to determine whether a system is stable, controllable and observable and, if not, to extract conditions such that it can be so. Such questions and tools to address them lie at the core of dynamical and control systems theory^4^. Furthermore, recent advances in sensing and actuation technology as well as the refinement of network models combined with increased computational capabilities, which allow us to better process and represent the plethora of data stemming from CDNs, have brought the fields of network science and control theory together and created a collection of novel and interesting problems. One of those problems is the driven node selection which consists of finding an optimal set of nodes to be driven such that the system attains certain properties. The optimality criterion is usually quantified by a combination of the number of driven nodes and/or the required control energy and the property to be attained is the controllability.

There have been many variants of the aforementioned problem and different heuristics have been devised to address its combinatorial nature. A prominent example on the controllability of CDNs was presented by Liu et al.^5^ where the authors used the Kalman’s rank condition and the idea of structural controllability^6^ to solve the minimum number of driving nodes (i.e., the nodes that provide the external input signal to different state nodes) by solving a maximum bipartite matching. The minimum number of driven nodes ensuring structural controllability^7^ and related problems have been solved by considering minimum input/output actuating costs^8^. Other variants include minimum structural perturbations^9^, constraints on the set of controlled states^10^, edge dynamics^11,12^, by introducing the metric of structural permeability^13^ to account for physical and economic constraints on the network and by accounting for the time-to-control^14^.

A limitation of these approaches is that they focus exclusively on structural controllability which may be a rather crude and misleading metric in some cases, as suggested by empirical findings in the field of cellular reprogramming^15^. This indicates that a more pertinent strategy is to determine the minimum number of driven nodes that optimizes a real-valued energy (e.g., controllability) metric. Gramian-based metrics^4^ have been extensively used^16–19^ to quantify the controllability of a complex network as they are related to the energy required to move the system in the state-space. An additional benefit of using Gramian-based metrics is that they possess the submodularity property^20^, which allows us to approximate the solution to the problem for large-scale systems using greedy methods. In this line of work, the proposed approaches include convex relaxations while accounting for rank constraints^21^, upper bounds on the control effort^22^ and joint optimization of performance and controllability using matroid constraints^23^.

One particularly important application of the controllability of complex dynamical networks is in the emerging field of controlling the human brain. It has been recently observed^24^ that cognitive brain control is analogous to mathematical notions of control used in engineering. The architecture of the brain, consisting of billions of neurons (nodes) interlinked by trillions synapses (edges), has a strong impact on neural function, brain development disease progression and rehabilitation. At a higher level, these neurons are organized in several anatomical regions (Fig. 4a), each of which is responsible for controlling a certain cognitive function such as memory and emotional expression (frontal lobe), motor control and movement (motor cortex) and auditory language and speech comprehension (temporal lobe). It is plausible^24^ that the brain could regulate cognitive function by transient network-level input through a process similar to the one used in control of complex networks. One important finding is that the brain network can be theoretically controllable by a single region/node, i.e., the smallest (in absolute value) of the eigenvalues of the controllability Gramian^24^. However, in order to assess the controllability of the brain network from one node, it is not necessary to compute the minimum eigenvalue, which in many practical cases admits extremely low values rendering the required control energy extremely high^25^. Alternatively, one can assess the controllability by proving the existence of a Hamiltonian path from each control region^26^, which however is a known NP-hard problem. The main pitfall of the current approaches is that they try to achieve controllability using a single node and that they ignore the long-term memory effects appearing in brain networks^27,28^.

While there has been a great effort to determine the minimum number of driven nodes in CDNs, most of the work focuses on Markovian (memoryless) or integer order dynamics and not much is known for the case where the dynamics are characterized by long-term memory. Long-term memory is captured by the notion of fractional derivative^29^ and many authors have shown that it is a more appropriate operator to describe the dynamics observed in real-world systems^30–32^. Even though classical control theoretic notions have been extended to fractional systems^29^, energy based methods for driven node selection are still in their infancy^33^.

In this paper, we introduce a fractional order system as a model for CDNs that exhibit long-term memory. Our model exploits fractional calculus concepts and generalizes all state-of-art models for CDNs. We present the controllability matrix for those networks, analyze the differences compared to integer order systems and extend this notion to a more sophisticated, real-valued, energy based metric that depends on the controllability Gramian. We exploit the structure of the latter objective function to design a computationally efficient greedy algorithm with approximation guarantees for the minimum number of driven nodes under energy requirements problem. Our method is applied on networks generated from the Erdős–Rényi, Barabási–Albert and Watts–Strogatz models. The effect of topology, parameters and model as well as the effect of memory on the number of driven nodes and controlled energy are assessed through comprehensive numerical simulations. Additionally, we show how our novel framework is applicable to the emerging field of brain controllability and we observe that certain brain cortex regions have a stronger impact than others.

## Results

### Experiments on complex network models

**Figure 1:**
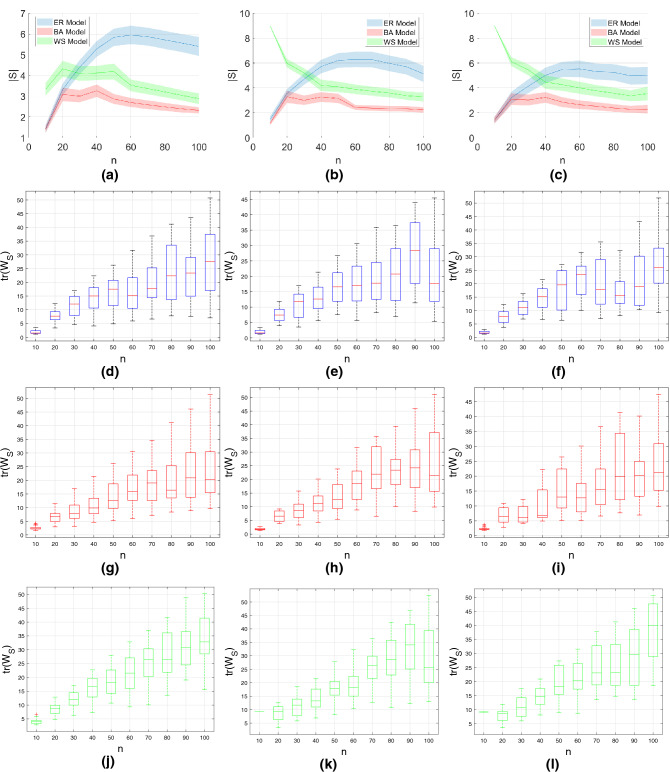
Investigating the effect of network on the number of driven nodes and Gramian trace. We use homogeneous order exponents of $\alpha =1$ (left column), $\alpha =0.5$ (middle column) and $\alpha =0.3$ (right column) and set the model parameters as follows: $p=0.5$ for the ER model; $m=4$ and $m_0=5$ for the BA model; $k=4$ and $\beta =0.8$ for the WS model. We run all simulations $N_r=50$ times and report the first and second order statistics. (**a**–**c**): Mean and $95\%$ confidence interval under the *t*-distribution assumption of the minimum number of driven nodes. (**d**–**f**) Quartiles and outliers of the trace of the controllability Gramian for the ER model. (**g**–**i**) Quartiles and outliers of the trace of the controllability Gramian for the BA model. (**j**–**l**) Quartiles and outliers of the trace of the controllability Gramian for the WS model.

We investigate the effects of topology and long-term memory on the number of driven nodes and the trace of the controllability Gramian in the context of Fractional Order Complex Dynamical Networks (FOCDNs) where the adjacency matrix is given by three different well-known models: the Erdős–Rényi (ER) model, the Barabási–Albert (BA) model and the Watts–Strogatz (WS) model. The edges are obtained by executing the corresponding network generation algorithm and a random weight drawn from the standard normal distribution is assigned. These models cover a wide spectrum of real-world complex networks as they capture fundamental topological properties such as random interactions (ER model), power-law degree distributions and preferential attachment based growth mechanisms (BA model), and small-world properties such as short path length and high clustering (WS model). In more detail, the ER model is characterized by the parameter *p* which is the connection probability of two randomly chosen nodes. Additionally, the BA model is constructed by starting with an initial, random connected network of $m_0$ nodes and connecting each newly added node to $m\le m_0$ existing nodes with a probability that is proportional to the number of links that the existing nodes already have. Finally, a WS graph is generated by initially creating a ring lattice with *N* nodes of mean degree 2*k*. Each node is connected to its *k* nearest neighbors on either side. Then, for each edge in the graph, we rewire the target node with probability $\beta$.

#### Effects of network size

To study the effect of network size, we set the model parameters as follows: $p=0.5$ for the ER model; $m=4$ and $m_0=5$ for the BA model; $k=4$ and $\beta =0.8$ for the WS model. We consider the memoryless case ($\alpha =1$ for all nodes) and the case where all nodes exhibit the same amount of memory (i.e., homogeneous fractional order exponents, $\alpha =0.5$ and $\alpha =0.3$). The optimal number of driven nodes as well as the trace of the Gramian upon algorithm termination are shown in Fig. 1. Observe that the ER model gives networks that are harder to control in terms of number of required driven nodes. The confidence intervals for all models are approximately steady for all investigated network sizes. The trace of the Gramian increases linearly with the network size for all models which implies that larger networks require higher control energy. Observe that the trace for the ER model is higher compared to either the BA or WS model implying that the former one is harder to control in terms of energy as well. We note that we performed as sensitivity analysis on the model parameters and obtained results similar to the above. This indicates that the effect of network size on the controllability is not dependant on the model parameters.

**Figure 2:**
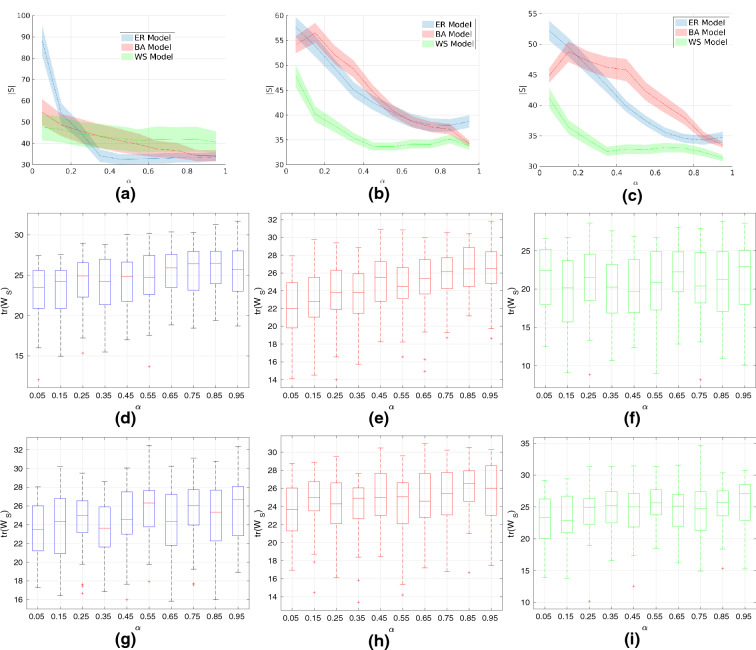
Effect of long-term memory on the number of driven nodes and Gramian trace. The network size is $N=100$ and the model parameters as follows: $p=0.5$ for the ER model; $m=4$ and $m_0=5$ for the BA model; $k=4$ and $\beta =0.8$ for the WS model. We run all simulations $N_r=50$ times and report the first and second order statistics. (**a**): Mean and 95% confidence interval under the *t*-distribution assumption of the minimum number of driven nodes for heterogeneous fractional order exponents. (**b**): Mean and 95% confidence interval under the *t*-distribution assumption of the minimum number of driven nodes for uniformly distributed fractional order exponents. (**c**) Mean and 95% confidence interval under the *t*-distribution assumption of the minimum number of driven nodes for normally distributed fractional order coefficients. (**d**–**f**) Quartiles and outliers of the trace of the controllability Gramian for uniformly distributed fractional order exponents. (**g**–**i**) Quartiles and outliers of the trace of the controllability Gramian for normally distributed fractional order exponents.

**Figure 3:**
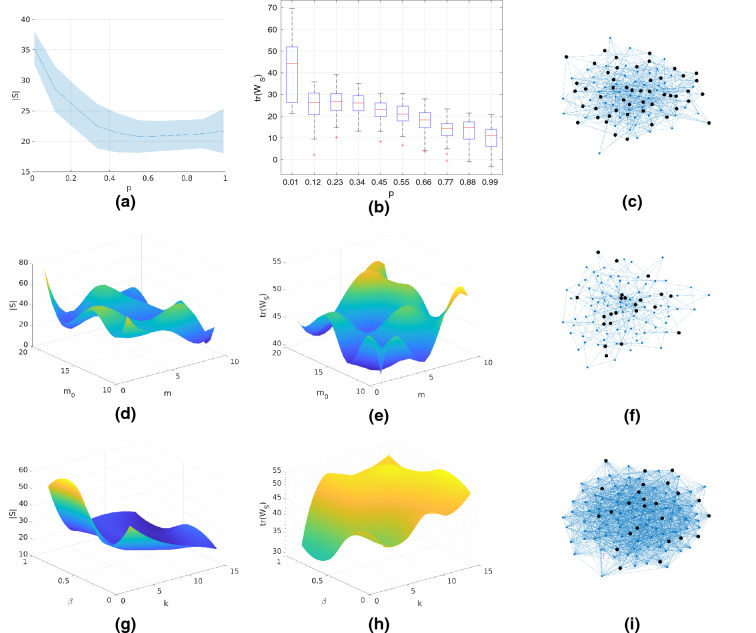
Investigating the effects of model parameters on the number of driven nodes and Gramian trace. We use networks of size $N=100$, Gaussian fractional order exponents, run all simulations $N_r=50$ times and report the first/second order statistics. (**a**) Mean and 95% confidence interval under the *t*-distribution assumption of the number of driven of the ER model. (**b**) Quartiles and outliers of the trace of the controllability Gramian for the ER model. (**c**) Sample graph from the ER model ($p=0.5$). The algorithms selected 50 nodes to be driven (highlighted). (**d**) Mean of the minimum number of driven nodes for the BA model. (**e**) Mean of the trace of the controllability Gramian for the BA model. (**f**) Sample graph from the ER model ($m=5,m_0=15$). The algorithms selected 24 nodes to be driven (highlighted). (**g**) Mean of the minimum number of driven nodes for the BA model. (**h**) Mean of the trace of the controllability Gramian for the BA model. (**i**) Sample graph from the WS model ($k=5,\beta =0.8$). The algorithms selected 25 nodes to be driven (highlighted).

#### Effects of long-term memory

We study the effect of long-term memory profiles (i.e., different distributions for the fractional order exponents) on the number of driven node and average control energy. We use networks of size $N=100$ and we investigate three different long-term memory profiles. For the first profile, we assume homogeneous fractional order which implies that all nodes exhibit the same amount of memory. We discretize the interval (0, 1] into a set of points $\{\alpha _i\}_{i=1}^{10}$ and interpolate the results. Then, we slightly vary the exponent of each node by drawing samples from a uniform (second profile) or a Gaussian distribution (third profile) of a small variance. Formally, if the fractional exponent of a node is $a_i$ in the first case, then it is $a_i+X$ where $X\sim U(-0.05,0.05)$ in the second case and $X\sim N(0,0.1^2)$ in the third case. The obtained results are shown in Fig. 2 and we observe that the existence of memory makes the network harder to control especially in terms of the number of the required driven nodes. More precisely, we see that, for different values of homogeneous order exponents, the number of driven nodes does not change significantly for the BA and WS models. On the contrary, when slightly varying the fractional exponent of each node around the mean we observe that the behavior changes significantly for all three models. More specifically, for higher values of the mean the network becomes easier to control as the number of needed driven nodes declines rapidly. The distribution from which the variations of the exponents are drawn does not have any significant effect. The fact that the variance around the mean is rather small indicates that this holds for a larger class of distributions. Regarding the trace of the Gramian, we observe that it does not change significantly for different long-term memory profiles, which indicates that the average control energy is not dependent on the fractional order exponents. Furthermore, the ER and BA appear to be slightly harder to control in terms of the average control energy as the trace of the Gramian is higher compared to the WS model.

#### Effects of model parameters

We investigate how the model parameters affect the controllability of the network. We use constant network size of $N=100$ nodes and assume that there exists Gaussian distributed, heterogeneous fractional order exponents. In more detail, the exponent $\alpha _i$ of each node is drawn from a Gaussian distribution centered around 0.5, i.e $\alpha _i \sim N(0.5,0.2)$. In the rare event that the exponent does not lie within the allowed interval (0, 1], it is re-sampled. We discretize the parameter space of the network models and interpolate the results which are shown in Fig. 3. For the ER model, we see that as the network becomes more dense (higher *p*) it requires lower number of driven nodes and a lower amount of control energy. A similar observation can be made for the BA model. On the contrary, we see that the more dense a WS network becomes the harder it gets to control it. This indicates that, in the presence of long-term memory, the effect of topology changes radically for WS networks compared to ER and/or BA networks.

### Controlling complex brain networks

**Figure 4:**
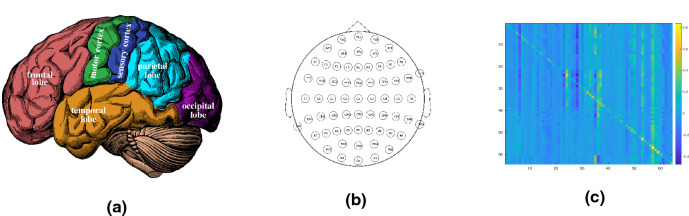
(**a**) Illustration of main anatomical brain regions (figure reproduced from^34^). (**b**) The position of the 64 EEG sensors used in the experimental study. (**c**) Resulting adjacency matrix of the extracted complex brain network from a randomly chosen subject.

In this section, we investigate how the topological features of a brain network affect our ability to steer it between states and therefore control its cognitive dynamics. Controllability of a brain network refers to the ability to manipulate the network components to drive the system along a desired trajectory with the purpose of reaching a target state, usually chosen for its functional utility. We define a *trajectory* of a neural system to be the temporal path that the system traverses through diverse states, where a *state* is defined as the magnitude of neurophysiological activity across brain regions at a single time point. Electroencephalogram (EEG) enables the neurophysiological monitoring of space-averaged synaptic source activity from millions of neurons. The existence of long-term memory in the neurophysiological activity of the brain is a widely debated topic and recent results tend to verify this hypothesis. In fact, fractional order models have been used for modeling^35^ and classification^36^ of EEG signals and demonstrated superior performance than their non-fractional counterparts.

**Figure 5:**
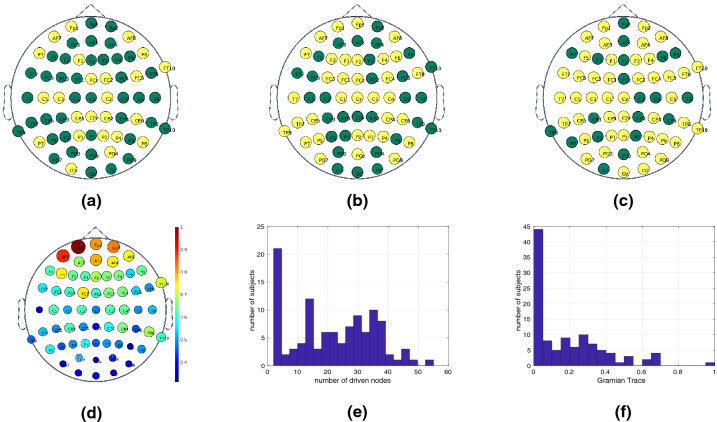
Brain controllability analysis. Highlighting in yellow the driven nodes required to achieve brain controllability for several individuals, namely individuals with ID 3 (in (**a**)), 27 (in (**b**)), and 102 (in (**c**)), respectively. The color-map representing the relative number of times each electrode acts as a driven node across all 109 subjects is shown in (**d**). The histogram of the number of driven nodes (**e**) and the trace of the Gramian (**f**) show that most of the subjects require few driven nodes (e.g., more than 20% of the subjects require less than 5 driven nodes) and a small fraction of 2 subjects that require more than 50 driven nodes.

A pivotal part in the analysis, design and control of complex brain networks using such fractional order models is the optimal parameter identification. In this experimental study, we use the model of Eq. (1), EEG data from the BCI competition^37^, a maximum likelihood based estimator for inferring the model parameters (i.e., adjacency matrix *A* and vector of fractional order exponents $\alpha$)^34,38^ as we leverage our novel framework to study the controllability of the brain. Our experimental setup consists of 109 subjects. We took the recorded 64-channel EEG signal, with the electrode distribution shown in Fig. 4b. The subjects were asked to perform motor and imagery tasks. The data was collected by BCI2000 system with sampling rate of 160 Hz^39^. After extracting the matrix *A* (an example is shown in Fig. 4c) and the fractional order exponent vector $\alpha$, we use our method to identify the driven nodes to ensure controllability of the complex brain network. Specific information about the EEG is provided in the Methods section.

Our results are summarized in Fig. 5. We plot the positions of the EEG electrode along with the driven nodes, as given by our method, for 3 randomly chosen subjects, i.e., subjects with id numbers 3 (Fig. 5a), 27 (Fig. 5b) and 102 (Fig. 5c). We observe that there is high inter-subject variability on the number of driven nodes, their positions as well as the brain areas that are activated. Which is consistent with our understanding of human brain networks, whereby neural activity on a certain brain region may affect the activity on a distant one through the action potentials that propagate within the neurons. The fact that there are less and more sparsely located driven nodes (subject 100) indicates that the underlying subject exhibits stronger connections between the different brain regions.

Another important observation is that in all three subjects it seems that there exist commonly driven nodes, i.e. the electrodes in the top part of the frontal lobe are driven in all three subjects. To verify this observation, we count the number of times that each node is driven across all 109 subjects and we plot the results (Fig. 5d). Observe that the $F_{P1}$ sensor lead (dark red color, top left) is driven in all subjects. Similar observations can be made for surrounding leads in the frontal lobe region, where as leads located at the occipital lobe are less commonly driven. This not only verifies the observation that we made on the three plotted subjects but it more importantly indicates that the frontal lobe region has a higher impact on the controllability of the brain. In fact, the leads that are driven in at least $80\%$ of the subjects are the $F_{P1}$, $AF_7$, $AF_Z$, $F_{PZ}$ and $F_{P2}$, all of which are located in the frontal lobe region.

To assess the performance of our algorithm, we plot the histograms of the number of driven nodes (Fig. 5e) and the normalized trace of the Gramian (Fig. 5f). The two combined metrics can be seen as a measure of the “energy” required to achieve brain controllability. We observe that more than $20\%$ of the subjects required less than 5 driven nodes with the rest being approximately uniformly distributed between 10 and 40. We also note that there exist few subjects who require high number of driven nodes ($>50$). Finally, the normalized trace of the Gramian exhibits a power-law behavior since almost half of the values are concentrated near zero. Both of these observations indicate that the set of driven nodes given by our algorithm archives relative low control energy which empirically validates its performance.

## Discussion

When controlling complex dynamical networks with long-term memory, we are interested in steering the system to a desired state while minimizing a mutli-objective criterion consisting of the number of driven nodes and the required control energy. We empirically analyzed a wide spectrum of network models and studied how the topological properties combined with long-term memory affect the number of driven nodes and the control energy. Regarding the network size, we observed that random networks are harder to control in terms of the number of driven nodes. Scale-free and small-world networks require lower number of driven nodes which indicates that their complex construction mechanism creates a topology that is controllable by injecting input signals to very few nodes. Furthermore, we studied how the parameters of the network models affect the controllability. Random and scale-free networks seem to exhibit a similar trend relating density and required control effort; the more dense the network the lower the required effort (number of driven nodes and control energy).

In addition to the above, we investigated how different long-term memory schemes affect the controllability of the network. We observed that heterogeneous order exponents make the network harder to control compared to the case where there is no memory. However, there appears to be no significant variation on the number of control nodes for different values of heterogeneous order exponents. On the contrary, the network is more difficult to control for lower mean values of uniformly or Gaussian distributed non-heterogeneous order exponents but the difficulty declines and converges to the heterogeneous case as the mean approaches 1. These results suggest that the existence of identical long-term memory profiles on the nodes render the network harder to control. Nonetheless, when there exist variations on the fractional order exponents (i.e., non-heterogeneous), the network is easier to control.

It would be reasonable to assume that the fractional exponents appearing in a real-world network are not identical for all nodes due to divergent physical, social or economic processes that characterize the dynamics of each node. Hence, our results on the memory effects imply that such networks may be prone to attacks. However, since we showed that heterogeneous order networks are harder to control, this paves the way to new research directions in an attempt to control CDNs and make them robust and resilient to adversarial perturbations. An important question that one may pose is how we could influence the fractional order exponents to drive a heterogeneous network to a non-heterogeneous such that the network is easier to control.

We showed how our method could be applied to the emerging field of brain control. We observed that some subjects required a high number of more densely located driven nodes, which could be due to them having weak neural connections between brain regions, which we hypothesize that may be a result of a neurodegenerative disease (e.g Alzheimer’s disease or brain trauma). We also noted that certain brain regions (such as the frontal lobe) have higher effect on the controllability of the entire network. We empirically validated the performance of our greedy algorithm since the combined energy metric (i.e. number of driven nodes and Gramian trace) has relatively low values for most of the subjects. Finally, one potential extension of our current work is the observability of CDNs. In fact, due to the known duality between controllability and observability^40^, our method is directly applicable (with minor changes in notation) to the problem of sensor selection to ensure observability.

## Methods

### Controllability and driven nodes in fractional-order complex dynamical networks (all simulations we run on Matlab 2020a)

A simple topological description of a CDN is a *graph* $\mathscr {G} = (V,E)$, where *V* is the set of *n* nodes and $E\subseteq V\times V$ is the set of edges. Two nodes $v,u\in V$ are *neighbors* if $(v_i,v_j)\in E$ and if, in addition, $(v_j,v_i)\in E$ then the graph is said to be *undirected*. A convenient description of a graph is given by the *weighted adjacency matrix* $A\in \mathscr {R}^{n\times n}$ where the *weight* $A_{ij}$ represents the strength of interaction between nodes $v_i,v_j\in V$. Each node $v_i\in V$ is associated with a dynamical *state* $x_i[k]\in \mathscr {R}$ which evolves in discrete time $k\in \mathscr {N}_{+}$ influenced by the interactions of its neighbors. We model the temporal evolution of the entire network via the following fractional-order complex dynamical network (FOCDN)

1 $$\begin{aligned} \Delta ^\alpha x[k+1] = A x[k] + B_Su[k] \end{aligned}$$

where $x[k] = (x_1[k],x_2[k], \ldots x_n[k])^T\in \mathscr {R}^N$ is a vector containing the states of all the nodes in the network at time *k*, $x[0]= x_0$ is a given initial state, $u[k]\in \mathscr {R}^m$ is the value of the *m*-dimensional control input signal injected in the network at time *k* and $\alpha \in (0,1]^n$ is a vector of fractional order exponents. The input matrix $B_S\in \mathscr {R}^{n\times m}$ identifies the nodes $S\subseteq V$ that are driven by a control input signal. In more detail, $B_S=diag\{b_1,b_2,\cdots ,b_n\}$ where $b_i=1$ if the node $v_i$ belongs to the set of driven nodes *S* and $b_i=0$ otherwise. The operator $\Delta ^\alpha$ denotes the Grünwald-Letnikov discretized derivative of order $\alpha = (\alpha _1,\cdots ,\alpha _n)$, which models the effects of temporal, long-term memory in the network dynamics^29^. If all fractional order coefficients are equal then this is referred to as a *homogeneous* order system, otherwise it is a *heterogeneous* order system. When the fractional order exponents approach 1 the system tends to become memoryless and when they are equal to 1 this model reduces to the commonly used one for CDNs^5^. On the contrary, when these coefficients approach zero the system exhibits far more pronounced memory, meaning that past states have a greater effect on future states. For each node *i* the Grunwald–Letnikov operator is defined as:

2 $$\begin{aligned} \Delta ^{\alpha _i}x[k+1] = x[k+1] + \sum _{j=1}^{k+1}(-1)^j {\alpha _i \atopwithdelims ()j}x[k-j+1] \end{aligned}$$

Let us define

3 $$\begin{aligned} A_0 = A - (c_1+1)I_n \quad A_j = diag\{c^i_{j+1}, i=1,2, \ldots n\}, \end{aligned}$$

where $c_j = -(-1)^{j} {\alpha _i \atopwithdelims ()(j+1)}$, for $j=1,\cdots ,N$. Then, by substituting Eq. (2) into Eq. (1), taking into account Eq. (3) and rearranging terms, we obtain the following:

4 $$\begin{aligned} x[k+1] = \sum _{j=0}^k A_j x[k-j] + B_Su[k]. \end{aligned}$$

It is evident from this equation that the proposed FOCDN captures long-term memory effects. In many practical cases, we are interested in finding a set of nodes that we need to drive to achieve convergence of the FOCDN to a given state, which is equivalent to ensuring that the system is controllable. Formally, the system is *controllable* if, for every initial condition $x_0\in \mathscr {R}^N$, there exists a control input able to steer the system to any arbitrary final state $x_d\in \mathscr {R}^n$ in at most $K\in \mathscr {N}_{+}$ time steps. To quantify this systemic property, we introduce the *controllability matrix*

5 $$\begin{aligned} \mathscr {C}(A,B_S,\alpha ;K) = [G_0B_S\;\;\;G_1B_S\;\;\;G_2B_S\;\;\cdots \;\;G_KB_S], \end{aligned}$$

where

6 $$\begin{aligned} G_k = {\left\{ \begin{array}{ll} \sum \nolimits _{j=0}^{k-1}A_jG_{k-j-1} &{}k\ge 1\\ I_n &{}k = 0 \end{array}\right. } \end{aligned}$$

The system is controllable if and only if $rank(\mathscr {C}(A,B_S,\alpha ;K)) = N$^41^. In the case where all fractional order exponents are equal to 1 (i.e, $\alpha =(1,1, \ldots 1)^T$), it is well known that the rank of $\mathscr {C}(A,B_S,\alpha ;K)$ cannot increase for any $K \ge N$, as a result of invoking the Cayley-Hamilton theorem^4^. On the contrary, in the case of a general, heterogeneous fractional-order system, the rank of $\mathscr {C}(A,B_S,\alpha ;K)$ can increase for values of $K \ge N$. In other words, it is possible to reach the final state $x_d$ in a number of steps greater than *n*. This is due to the time-varying nature of the elements $G_k$ which build up the controllability matrix^29^. The full rank can be reached at some time step equal to or greater than *n*. This distinctive property of fractional order dynamics posses no real restriction as, in practice, we need to steer the system to the desired state within a given time frame. This implies that there exists a given upper bound on the value of *K*, which we can use to test for controllability. We now introduce the *controllability* (or *reachability*) *Gramian* defined as follows

7 $$\begin{aligned} W_S:=W(A,B_S,\alpha ;K) = \sum _{j=0}^{K-1} G_jB_SB_S^TG_{j}^T, \end{aligned}$$

where $G_k$ is defined as in Eq. (6). It can be verified that the reachability Gramian can be expressed in terms of the controllability matrix as follows $W_S=\mathscr {C}(A,B_S,\alpha ;K)\mathscr {C}^T(A,B_S,\alpha ;K)$. The equivalent controllability condition is $rank(W_S)=N$. The advantage of using the Gramian instead of the controllability matrix is that the former one provides an energy-related quantification of controllability^20^. Eigenvectors of $W_S$ associated with small eigenvalues (large eigenvalues of $W_S^{-1}$) define directions in the state space that are less controllable (require large input energy to reach). Conversely, eigenvectors of $W_S$ associated with large eigenvalues (small eigenvalues of $W_S^{-1}$) define directions in the state space that are more controllable (require small input energy to reach). Intuitively, we want $W_S$ “large” so that $W_S^{-1}$ is “small”, requiring small amount of input energy to move around the state space. Based on this observation, we can define the following scalar controllability metric

8 $$\begin{aligned} f(S) = tr(W_S). \end{aligned}$$

The trace of the controllability Gramian is inversely related to the average energy and can be interpreted as the average controllability in all directions in the state space. Note that *f*(*S*) depends on the set of driven nodes via matrix $B_S$ (Eq. 7). The problem is to choose this set $S\subseteq V$ to maximize the above metric while keeping the size of *S* as small as possible. This can be easily proved to be an *NP*-hard optimization problem. Nonetheless, the objective function given by Eq. (8) is modular allowing us to solve a *NP*-hard combinatorial optimization problem using a greedy algorithm with approximation guarantees. In more detail, we start with an empty set of driven nodes $S_0=\emptyset$, pick the node $v\in V$ whose addition to the set of driven nodes leads to highest gain in the value of the metric, add *v* in the set of driven nodes and repeat using the set of non-driven nodes $v\setminus S_0$. Under a cardinally constraint on the driven nodes set this greedy algorithm achieves an approximation ratio of 1/*e*, which is the best approximation that any polynomial algorithm can achieve, assuming that $P\ne NP$^42,43^. In our formulation we aim to achieve controllability, therefore we impose an additional rank constraint on the Gramian. This implies that the algorithm is terminated when the controllability Gramian reaches full rank.

#### Driven node selection in FOCDNs

Driven node selection in FOCDNs problems can be formulated as set function optimization problem. For the given finite set of nodes *V*, a set function $f:2^n \rightarrow \mathscr {R}$ assigns a real number to each subset $S\subseteq V$. In our setting, the elements of *S* represent potential nodes of the FOCDN which could be driven, and the function *f* is a metric for how controllable the system is for a given set of driven node selection. The problem can been seen as selecting the non-zero elements of the diagonal input matrix $B_S$ appearing in Eq. (1) such that the multi-objective function consisting of the number of driven nodes and the negative trace of the controllability Gramian is minimized and the system is controllable, i.e., given $(A,B,\alpha ,K)$ and for $k\in \{1,\ldots ,n\}$ determine the solution of the following problem:

9 $$\begin{aligned} &\underset{S\subseteq V, |S|=k}{\text {minimize}}&(k,-tr(W(A,B_S,\alpha ;K))) \\&\text {subject to}& rank(W(A,B_S,\alpha ;K)) = N. \end{aligned}$$

It can be easily proved that (9) is an *NP*-hard optimization problem. One alternative to find the solution to this problem is to consider a brute force approach that consists by enumerating all possible subsets of size *k*, evaluating the trace of the Gramian for all of these subsets, and picking the one that optimizes the multi-objective function. However, we are interested in cases arising from real-world complex networks in which the number of possible subsets is very large. The number of possible subsets grows factorially as the number of nodes increases, rendering a brute-force approach computationally infeasible. Therefore, we focus on exploiting structural properties of the objective function, particularly the submodularity property^20^.

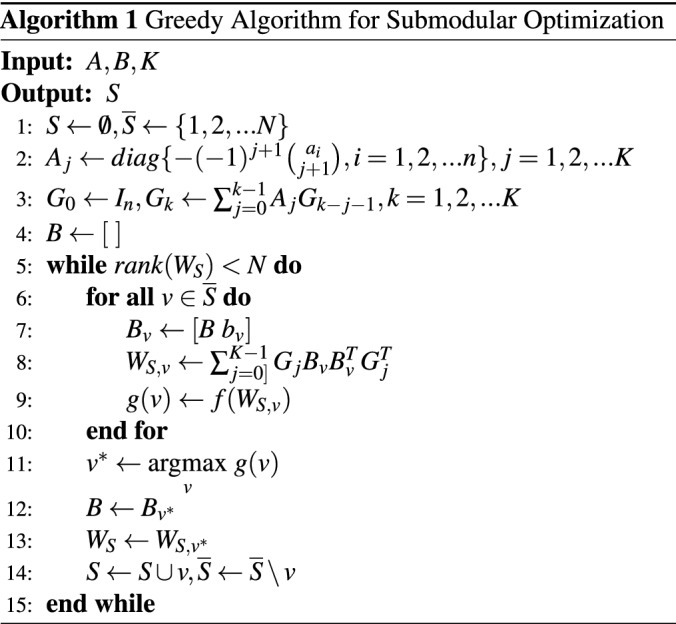



The set function $f:2^V\rightarrow \mathscr {R}$ is called *submodular* if for all subsets $A\subseteq B\subseteq V$ and all elements $s\not \in B$, it holds that $f(A\cup \{s\}) - f(A)\ge f(B\cup \{s\}) - f(B)$ or, equivalently, if for all subsets $A,B\subseteq V$, it holds that $f(A)+f(B) \ge f(A\cup B) +f(A\cap B)$. Intuitively, submodularity is a diminishing returns property where adding an element to a smaller set gives a larger gain than adding one to a larger set. In addition to that, it is called *monotone increasing* if for all subset $A,B\subseteq V$ it holds that $f(A)\le f(B)$ and *monotone decreasing* if for all subset $A,B\subseteq V$ it holds that $f(A)\ge f(B)$. Maximization of monotone increasing submodular functions is NP-hard, but the so-called greedy heuristic can be used to obtain a solution that is provably close to the optimal solution. The pseudo-code of the greedy algorithm for the problem described in Eq. (9) is given in Algorithm 1. The returned set of driven nodes *S* is associated with an objective value *f*(*S*) and its difference from the optimal objective value $f(S^*)$ given by the optimal set of driven nodes is guaranteed to be upper bounded as follows:

10 $$\begin{aligned} f(S^*) - f(S) = \frac{1}{e}(f(S^*) - f(\emptyset )), \end{aligned}$$

where $f(\emptyset )$ denotes the objective value when no node is driven. A major difficulty faced in the driven node selection problem stems from numerical instabilities in the estimation of the rank of a matrix. For the system to achieve controllability and, hence the greedy algorithm to terminate, the controllability Gramian needs to have full rank. However, even for moderately sized networks the rank computation gets computationally unstable quite rapidly. For example, experiments showed that if we consider a BA random graph of $N=50$ nodes, drive every node (i.e, the matrix *B* appearing in Eq. (1) is the identity matrix) and compute the eigenvalues of the controllability Gramian then the largest one is of order $10^{40}$ while the smallest one of order $10^{20}$. When compared to the largest ones, the smallest eigenvalues are considered numerically zero and, therefore, the rank computation, which gives a rank significantly lower than 50, incorrectly concludes that the system is uncontrollable.

The aforementioned issue is a direct consequence of an extremely high condition number (defined as the ratio of the largest to smallest eigenvalue) which results in an inaccurate rank estimation^44^. Since the size of an eigenvalues is proportional the energy required to steer the system in the direction of the corresponding eigenvector in the state space, by considering the modeling and application context when interpreting whether the required energy for state transfer is feasible we can appropriately threshold the eigenvalues of the Gramian to obtain a domain-specific estimation of the rank. To address this computational issue in our experiments, we considered the QR decomposition the controllability Gramian and estimated the rank as the number of diagonal elements of the R matrix that are above a certain threshold. The QR decomposition was selected because it produces matrices with lower condition number and a sub-optimal threshold was empirically found by testing several fully-driven networks. The reasoning that we used for finding that threshold is that fully-driven networks give rise to a controllability Gramian that is full rank (because the network is fully controllable). Therefore, by testing networks of different sizes and adjusting the threshold such that the resulting rank estimation is correct, we are able to obtain an empirical value.

Regarding the computational complexity of Algorithm 1, observe that the outer loop is executed at most *n* times due to the fact that if we drive all nodes then the network is controllable and, therefore, the Gramian is full rank. Also, the inner loop is executed at most *N* times as well which gives rise to a computational complexity of $\mathscr {O}(N^2)$. However, we note that an important practical restriction comes from the computation of the Gramian. Assuming that the chosen time horizon *K* is proportional to the size of the network and utilizing the Coppersmith–Winograd algorithm for matrix multiplication (which achieves a runtime of $\mathscr {O}(N^{2.374})$), we see from Eqs. (6) and (7) that the computation of the Gramian takes $\mathscr {O}(N^{5.748})$ time. This is partially due to the recursive nature of the state transition matrix $G_k$ given by Eq. (6). One potential way to reduce the time complexity is to reduce the time horizon to $K<<N$. This could reduce the time complexity by one order of magnitude but it would require the system to reach controllability in a shorter time frame, which in general is harder. Another way to reduce the complexity is to exploit the structure of the $A_j$ matrix, which is diagonal for $j\ge 1$ as seen in Eq. (3), to reduce the complexity of matrix multiplication to $\mathscr {O}(N^2)$. This reduces the complexity of the Gramian computation to $\mathscr {O}(N^5)$ and the overall complexity of the algorithm to $\mathscr {O}(N^7)$, which is impractical for large networks. Nonetheless, we emphasize that scaling the method to large networks is not bottle-necked by the performance of our algorithm but rather by numerical stability issues in computing the rank of the Gramian, which are well known in the case of integer-order systems^45,46^. These issues persist in the fractional order case as well, since we can retrieve the integer-order system as a particular case.

### EEG technology for brain activity monitoring: an overview^34^

The Electroencephalogram (EEG) enables us to monitor the spatial average of the synaptic activity on the neocortex, a part of the human brain involved in high-level functions such as sensory perception, cognition and reasoning. Even though the EEG signals as characterized by poor spatial resolution, due to physical limitations in placing sensors, they have high temporal resolution since the electrical activity from the neocortex reaches the recording site within milliseconds. The electrodes (i.e., sensors or leads) are placed over the area of interest on the scalp most commonly following the International 10–20 system, as depicted in Fig. 4. The rhythmic fluctuations occurring between inhibitory interneurons and excitatory pyramidic cells are known as oscillations. Even though the origin of such oscillations is not yet understood, the EEG electrodes are capable of capturing their activity. Furthermore, there exists evidence that the brain activity is processed at certain frequencies. Therefore, oscillatory behavior in the human brain is often partitioned in frequencies or bands (covering a wide range of frequencies decaying as 1/*f* in power): (i) $\delta$-band (0.5–3 Hz); (ii) $\theta$-band (3.5–7 Hz); (iii) $\alpha$-band (8–13 Hz); (iv) $\beta$-band (14–30 Hz); and (v) $\gamma$-band (30–70 Hz). Certain high-level brain functions such as sensory registration, perception cognitive processes related to attention, learning and memory are associated with brain activity in certain bands^47^. Different changes in the signals across different bands are also used to interpret the event-related potentials (ERPs) in the EEG signals, i.e., variations due to specific events^48^. These represent oscillations that are recorded over the posterior frontal and anterior parietal areas of the brain, i.e., over the sensorimotor cortices (see Fig. 4). SMRs occur mainly in the $\alpha$-band (for sensors located on the top of the motor cortices), and on $\beta$ and lower $\gamma$ for those on the sensorimotor cortices^49^.
